# Supplementary material for: The Evolution and Characterization of the RNA Interference Pathways in Lophotrochozoa
Source: Genome Biol Evol. 2024 May 7;16(5):evae098. doi: 10.1093/gbe/evae098 (PMC11114477; doi:10.1093/gbe/evae098)
Supplement: evae098_Supplementary_Data [file evae098_supplementary_data.zip › Supplementary_materials.zip!/selected_miRNAs/NoGe_selected_miRNAs/mirdeep_pdf_files/NMRB01000795.1_Notospermus_geniculatus_strain_Ushimado_scaffold795,_whole_genome_shotgun_sequence_20949.pdf]

Provisional ID	:	NMRB01000795.1_Notospermus_geniculatus_strain_Ushimado_scaffold795,_whole_genome_shotgun_sequence_20949
Score total	:	8955.6
Score for star read(s)	:	3.9
Score for read counts	:	8950.6
Score for mfe	:	1.7
Score for randfold	:	0
Score for cons. seed	:	-0.6
Total read count	:	17568
Mature read count	:	17478
Loop read count	:	0
Star read count	:	90

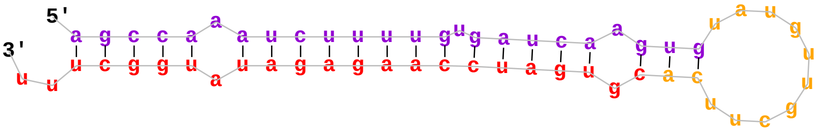

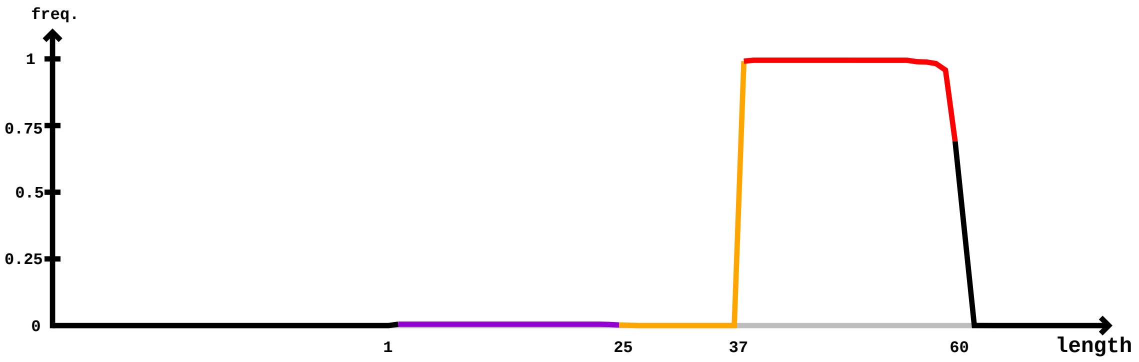


```
                                    Star                               Mature
5'- caaauccacaaguuccaaaagggguguguauccacagccaaaucuuuugugaucaaguguauguugcuucacgugauccaagagauauggcuuuggucaucacccccau   -3'   obs
    caaauccacaaguuccaaaagggguguguauccacagccaaaucuuuugugaucaaguguauguugcuucacgugauccaagagauauggcuuuggucaucacccccau         exp
    .....................(((.(((...(((.(((((.((((((((.(((((.(((..........))).)))))))))))))).))))).))).....))).))).. reads      mm      sample
    .......................................................................cgugauccGagagauauggcuu................   9          1       Fm2
    .......................................................................cgugauccaagagauauggcuuu...............   7          0       Fm2

    .......................................................................Agugauccaagagauauggcuuu...............   1          1       Fm1
    .......................................................................cgugauccaagagauauggcuuu...............   31         0       Fm1
    .......................................................................cgugauccGagagauauggcuuu...............   3          1       Fm1
    .......................................................................cgugauccaagagauauggcuuG...............   1          1       Fm1
    .......................................................................cgugauccaagagauauggcuuuU..............   6          1       Fm1

    ...........................................................uauguugcuucacgugauccaagU..........................   1          1       Mm2
    .......................................................................cgugauccaagagauaug....................   38         0       Mm2
    .......................................................................cgugauccaaUagauaug....................   1          1       Mm2
    .......................................................................cgugauccaagagauaugU...................   3          1       Mm2
    .......................................................................cgugauccaagagauaugg...................   6          0       Mm2
    .......................................................................cgugauccaagagauauggc..................   61         0       Mm2
    .......................................................................cgugauccaagagauauUgc..................   2          1       Mm2
    .......................................................................cguUauccaagagauauggc..................   1          1       Mm2
    .......................................................................cgugauccaagagauauggcu.................   227        0       Mm2
    .......................................................................cgugauccaaUagauauggcu.................   1          1       Mm2
    .......................................................................cgugauccGagagauauggcu.................   1          1       Mm2
    .......................................................................cguAauccaagagauauggcu.................   1          1       Mm2
    .......................................................................cgugauccaagagauauCgcu.................   2          1       Mm2
    .......................................................................cgugauccaagagauaCggcu.................   1          1       Mm2
    .......................................................................cgugauccaagagauaGggcu.................   1          1       Mm2
    .......................................................................cgugauccaagagauauUgcu.................   2          1       Mm2
    .......................................................................cgugauccaaCagauauggcu.................   1          1       Mm2
    .......................................................................cgugauccaagagauauggcG.................   2          1       Mm2
    .......................................................................cgugauccaagagauauAgcu.................   1          1       Mm2
    .......................................................................cgugauccaagagauauggcA.................   3          1       Mm2
    .......................................................................Ugugauccaagagauauggcu.................   1          1       Mm2
    .......................................................................cgugauccaagagauaCggcuu................   13         1       Mm2
    .......................................................................cgugaCccaagagauauggcuu................   1          1       Mm2
    .......................................................................cUugauccaagagauauggcuu................   1          1       Mm2
```

```
                                  Star                                Mature
caaauccacaaguuccaaaaggguguguauccacagccaaaucuuuugugaucaaguguauguugcuucacgugauccaagagauauggcuuuggucaucacccccau
......................................................................cgugauccaagagCuauggcuu................   2      1      Mm2
......................................................................cgugauccaagagauauggcuG................   8      1      Mm2
......................................................................Ngugauccaagagauauggcuu................   3      1      Mm2
......................................................................cgugauccaaUagauauggcuu................   4      1      Mm2
......................................................................cgugauccaagagauauggcuC................   1      1      Mm2
......................................................................cgugauccaagagaCauggcuu................   1      1      Mm2
......................................................................cgugauccaagagauGuggcuu................   2      1      Mm2
......................................................................cgugauccaagagauauCgcuu................   16     1      Mm2
......................................................................cgugauccaagagauauggUuu................   2      1      Mm2
......................................................................cgugGuccaagagauauggcuu................   1      1      Mm2
......................................................................cgugaucGaagagauauggcuu................   1      1      Mm2
......................................................................cgugauccaagagauauggcAu................   2      1      Mm2
......................................................................cgugauccaagagauauggcuu................   2695   0      Mm2
......................................................................cgugauccaagagauaGggcuu................   2      1      Mm2
......................................................................cgugauAcaagagauauggcuu................   1      1      Mm2
......................................................................cgugauccaaAagauauggcuu................   2      1      Mm2
......................................................................cgugauccaagagauauAgcuu................   19     1      Mm2
......................................................................cguCauccaagagauauggcuu................   1      1      Mm2
......................................................................cgugauccaagagauauUgcuu................   7      1      Mm2
......................................................................cgugauccaagagauaAggcuu................   6      1      Mm2
......................................................................cgugauccaagagauauggcuA................   2      1      Mm2
......................................................................cgugauccaaCagauauggcuu................   2      1      Mm2
......................................................................cgugauccCagagauauggcuu................   1      1      Mm2
......................................................................cgAgauccaagagauauggcuu................   1      1      Mm2
......................................................................cgugaucAaagagauauggcuu................   1      1      Mm2
......................................................................cgugauccaagagGuauggcuu................   1      1      Mm2
......................................................................cgugauccGagagauauggcuu................   5      1      Mm2
......................................................................cgugauccaagaUauauggcuuu...............   1      1      Mm2
......................................................................cgugauccaaAagauauggcuuu...............   14     1      Mm2
......................................................................cgugauccaagagCuauggcuuu...............   3      1      Mm2
......................................................................cgugauccaagagauaAggcuuu...............   17     1      Mm2
......................................................................cgugauAcaagagauauggcuuu...............   1      1      Mm2
......................................................................cgugaucUaagagauauggcuuu...............   5      1      Mm2
......................................................................cCugauccaagagauauggcuuu...............   1      1      Mm2
......................................................................cgugauccaaCagauauggcuuu...............   3      1      Mm2
......................................................................cgugauccaagagauGuggcuuu...............   4      1      Mm2
......................................................................cgugauccaagagaAauggcuuu...............   1      1      Mm2
......................................................................cAugauccaagagauauggcuuu...............   1      1      Mm2
......................................................................cgugauccaagagaGauggcuuu...............   3      1      Mm2
......................................................................cgugauccaagagauauggcuuG...............   5      1      Mm2
......................................................................cgugauccaagagauauAgcuuu...............   52     1      Mm2
......................................................................cgugauccaagaAauauggcuuu...............   2      1      Mm2
......................................................................cgugauccaUgagauauggcuuu...............   3      1      Mm2
......................................................................cgugauccaagagauauggcuCu...............   2      1      Mm2
......................................................................cgugaucGaagagauauggcuuu...............   1      1      Mm2
......................................................................cgugauccUagagauauggcuuu...............   1      1      Mm2
......................................................................cgugaucAaagagauauggcuuu...............   1      1      Mm2
......................................................................cgugauccaagagauaGggcuuu...............   15     1      Mm2
......................................................................cgugauccaagagauUuggcuuu...............   1      1      Mm2
......................................................................cgugauccaagagUuauggcuuu...............   2      1      Mm2
......................................................................cgugauccaagagauauggcuuC...............   3      1      Mm2
......................................................................cgugaCccaagagauauggcuuu...............   1      1      Mm2
......................................................................cgCgauccaagagauauggcuuu...............   3      1      Mm2
......................................................................Ngugauccaagagauauggcuuu...............   5      1      Mm2
......................................................................cgugauUcaagagauauggcuuu...............   2      1      Mm2
......................................................................cgugauccaagGgauauggcuuu...............   2      1      Mm2
......................................................................cUugauccaagagauauggcuuu...............   1      1      Mm2
......................................................................cgugauccaagagauauCgcuuu...............   42     1      Mm2
......................................................................Ugugauccaagagauauggcuuu...............   8      1      Mm2
......................................................................cguAauccaagagauauggcuuu...............   2      1      Mm2
......................................................................cgugauccaagCgauauggcuuu...............   1      1      Mm2
......................................................................cgugauccaagagauaCggcuuu...............   22     1      Mm2
......................................................................cgugauccaagagauauggcuuA...............   90     1      Mm2
......................................................................cgugauccGagagauauggcuuu...............   39     1      Mm2
......................................................................cgugauccaaUagauauggcuuu...............   14     1      Mm2
......................................................................cgugaNccaagagauauggcuuu...............   1      1      Mm2
......................................................................Ggugauccaagagauauggcuuu...............   1      1      Mm2
......................................................................cgugauccaagagauauggcuuu...............   7454   0      Mm2
......................................................................cgugauccaGgagauauggcuuu...............   2      1      Mm2
......................................................................cgugUuccaagagauauggcuuu...............   2      1      Mm2
```

```
                                  Star                               Mature
caaauccacaaguuccaaaaggguguguauccacagccaaaucuuuugugaucaaguguauguugcuucacgugauccaagagauauggcuuuggucaucacccccau
......................................................................cgugGuccaagagauauggcuuu...............  1    1  Mm2
......................................................................cgugauccaagagauauUgcuuu...............  30   1  Mm2
......................................................................cgugauccCagagauauggcuuu...............  1    1  Mm2
......................................................................cgugauccaagagauauggcuuuU..............  871  1  Mm2
......................................................................cgugauccaagagauauggcuuuC..............  2    1  Mm2
......................................................................cgugauccaagagauauggcuuuA..............  41   1  Mm2
......................................................................cgugauccaagagauauggcuuug..............  1    0  Mm2
......................................................................cgugauccaagagauauCgcuuug..............  1    1  Mm2
......................................................................cgugauccaagagauauggcuuugU.............  1    1  Mm2
......................................................................cgugauccaagagauauggcuuuAgu............  2    1  Mm2
.......................................................................gugauccaagagauauggc..................  1    0  Mm2
.......................................................................gugauccaagagauauggcuu................  6    0  Mm2
.......................................................................gugauccaagagauaugCcuuu...............  1    1  Mm2
.......................................................................gugauccaagagauauggcuuu...............  33   0  Mm2
.......................................................................gugauccaagagauauggcuuuU..............  2    1  Mm2

..................................agccaaaucuuuugugaucaag....................................................  14   0  Mm1
..................................agccaaaucuuuugugaCcaag....................................................  1    1  Mm1
..................................agccaaaCcuuuugugaucaag....................................................  1    1  Mm1
..................................agccaaaucuuuugugaucaagu...................................................  36   0  Mm1
..................................agccaaaucuuuugugaucaaguU..................................................  4    1  Mm1
..................................agccaaaucuuuugugaucaagug..................................................  33   0  Mm1
..................................agccaaaucuuuugCgaucaagug..................................................  1    1  Mm1
......................................................................cgugauccaagagauaug....................  39   0  Mm1
......................................................................cgugauccaagagauaugU...................  9    1  Mm1
......................................................................cgugauccaagagauauggc..................  22   0  Mm1
......................................................................Ugugauccaagagauauggc..................  1    1  Mm1
......................................................................cgugauccaagagauauggcu.................  80   0  Mm1
......................................................................cgugauccaagagauauAgcu.................  1    1  Mm1
......................................................................cgugauccaagagauaAggcuu................  5    1  Mm1
......................................................................cgugauccaaCagauauggcuu................  1    1  Mm1
......................................................................cgugauccaagagGuauggcuu................  1    1  Mm1
......................................................................cgugauccaagagauauAgcuu................  2    1  Mm1
......................................................................cgugauccaagagauauCgcuu................  7    1  Mm1
......................................................................cguAauccaagagauauggcuu................  1    1  Mm1
......................................................................cgugauccaagagauGuggcuu................  2    1  Mm1
......................................................................cgugaucUaagagauauggcuu................  1    1  Mm1
......................................................................cgugauccaagagauauggcuu................  836  0  Mm1
......................................................................cgugauccCagagauauggcuu................  1    1  Mm1
......................................................................cgugauccaagagauaGggcuu................  1    1  Mm1
......................................................................cgugauccaagagauaCggcuu................  2    1  Mm1
......................................................................cgugauccaagagauauUgcuu................  6    1  Mm1
......................................................................Ngugauccaagagauauggcuu................  1    1  Mm1
......................................................................Ugugauccaagagauauggcuu................  1    1  Mm1
......................................................................cgugauccaagUgauauggcuuu...............  1    1  Mm1
......................................................................cgugauccaagagauaAggcuuu...............  1    1  Mm1
......................................................................cgugauccaagagauauggcuuu...............  928  0  Mm1
......................................................................cgugauccaagagauauggcuuA...............  52   1  Mm1
......................................................................cgugaNccaagagauauggcuuu...............  1    1  Mm1
......................................................................cgugauccaaAagauauggcuuu...............  3    1  Mm1
......................................................................cgugauUcaagagauauggcuuu...............  3    1  Mm1
......................................................................cgugaucUaagagauauggcuuu...............  2    1  Mm1
......................................................................cgugauccaagagauauggcuuG...............  1    1  Mm1
......................................................................cgugauccaagagauauAgcuuu...............  4    1  Mm1
......................................................................cgugauccaagagauaGggcuuu...............  1    1  Mm1
......................................................................cgCgauccaagagauauggcuuu...............  1    1  Mm1
......................................................................cgugauccaagagauauUgcuuu...............  7    1  Mm1
......................................................................cgugauccaagagauauCgcuuu...............  2    1  Mm1
......................................................................cgugauccaagagauaCggcuuu...............  2    1  Mm1
......................................................................cgugauccaagagauGuggcuuu...............  4    1  Mm1
......................................................................cgugauccGagagauauggcuuu...............  18   1  Mm1
......................................................................cgugauccaagagauauggcuuuA..............  28   1  Mm1
......................................................................cgugauccaagagauauggcuuuU..............  59   1  Mm1
.......................................................................gugauccaagagauauggcuuu...............  6    0  Mm1

......................................................................cgugauccaagagauauggcu.................  8    0  Fm3
......................................................................cgugauccaagagauauggcuu................  7    0  Fm3
......................................................................Ggugauccaagagauauggcuuu...............  1    1  Fm3
......................................................................cgugauccaagagauauggcuuA...............  1    1  Fm3
......................................................................cgugauccaagagauauggcuuu...............  8    0  Fm3
```

Star **Mature**

caaauccacaaguuccaaaaggguguguauccacagccaaaucuuuugugaucaaguguauguugcuucacgugauccaagagauauggcuuuggucaucacccccau

....cgugauccaagagauauU....................	1	1	Mm3
....cgugauccaagagauaug....................	17	0	Mm3
....cgugauccaagagauaugU...................	1	1	Mm3
....cgugauccaagagauauggc..................	14	0	Mm3
....cgugauccaagagauauggcC.................	1	1	Mm3
....Agugauccaagagauauggcu.................	1	1	Mm3
....cgugauccaaCagauauggcu.................	1	1	Mm3
....cgugauccaagagauauggcA.................	5	1	Mm3
....cgugauccaagagauaGggcu.................	1	1	Mm3
....cgugauccaagagauauggcu.................	84	0	Mm3
....cgugauccaagagauauAgcu.................	1	1	Mm3
....cgugauccaagagauauUgcu.................	1	1	Mm3
....Ngugauccaagagauauggcu.................	1	1	Mm3
....cgugauccaagagauauggcG.................	1	1	Mm3
....cgugauccaagagauaAggcuu................	1	1	Mm3
....cgugauccaagagauCuggcuu................	1	1	Mm3
....cgugauccaaAagauauggcuu................	2	1	Mm3
....cgugauccaagagauaugUcuu................	2	1	Mm3
....cgugauccaagagauauggcCu................	1	1	Mm3
....cUugauccaagagauauggcuu................	1	1	Mm3
....cgugauccaagagauaGggcuu................	3	1	Mm3
....cgugauccaagagauauUgcuu................	3	1	Mm3
....cgugauccaaCagauauggcuu................	1	1	Mm3
....cgugauccaagagauauggcuu................	965	0	Mm3
....cgugauccaagagauauCgcuu................	7	1	Mm3
....cgugauccGagagauauggcuu................	8	1	Mm3
....cgAgauccaagagauauggcuu................	1	1	Mm3
....cgugauccaagagauauggcuG................	1	1	Mm3
....cgugauccaagagauauAgcuu................	3	1	Mm3
....cgugauccaagagaCauggcuuu...............	1	1	Mm3
....cgugaCccaagagauauggcuuu...............	1	1	Mm3
....cgugauccaagagauauCgcuuu...............	9	1	Mm3
....Ugugauccaagagauauggcuuu...............	1	1	Mm3
....cgugauccaagagauaCggcuuu...............	4	1	Mm3
....cgugauccaaAagauauggcuuu...............	2	1	Mm3
....cgugauccaaCagauauggcuuu...............	3	1	Mm3
....cgugauccaagagauauggcuuA...............	48	1	Mm3
....cgugauccaagagauauUgcuuu...............	9	1	Mm3
....Ngugauccaagagauauggcuuu...............	1	1	Mm3
....cgugauccaagagauauggcuuu...............	1743	0	Mm3
....cgugauccaagagauauAgcuuu...............	16	1	Mm3
....cgugauccaagaUauauggcuuu...............	1	1	Mm3
....cgugauccaagagauaAggcuuu...............	3	1	Mm3
....cgugauccaagagauauggcuuC...............	1	1	Mm3
....cguUauccaagagauauggcuuu...............	1	1	Mm3
....cAugauccaagagauauggcuuu...............	1	1	Mm3
....cgugauccaagUgauauggcuuu...............	2	1	Mm3
....cguCauccaagagauauggcuuu...............	1	1	Mm3
....cgugauccGagagauauggcuuu...............	16	1	Mm3
....cgugauccaagagauaGggcuuu...............	4	1	Mm3
....cgugauccaagagauauggcuuG...............	2	1	Mm3
....cgugauccaagagauauggcuuuC..............	1	1	Mm3
....cgugauccaagagauauggcuuug..............	1	0	Mm3
....cgugauccaagagauauggcuuuA..............	23	1	Mm3
....cgugauccaagagauauggcuuuU..............	228	1	Mm3
....gugauccaagagauauggcu.................	1	0	Mm3
....gugauccGagagauauggcuuu...............	1	1	Mm3
....gugauccaagagauauggcuuu...............	7	0	Mm3
....gugauccaagagauauggcuuuU..............	1	1	Mm3
